# Prenatal diagnosis of a compound heterozygous variation in the *FBXL4* gene by trio-WES and imaging monitoring: a case report

**DOI:** 10.3389/fgene.2025.1539288

**Published:** 2025-04-25

**Authors:** Yujia Zhai, Jing Chen, Shuo Yang, He Wang, Yuanyuan Xiao, Shanling Liu

**Affiliations:** ^1^ Department of Medical Genetics/Prenatal Diagnostic Center, West China Second University Hospital, Sichuan University, Chengdu, China; ^2^ Key Laboratory of Birth Defects and Related Diseases of Women and Children (Sichuan University), Ministry of Education, Chengdu, China

**Keywords:** prenatal diagnosis, FBXL4, NT thickening, MTDPS13, trio-WES

## Abstract

F-box and leucine-rich repeat protein 4 (FBXL4) plays a crucial role in mitochondrial bioenergetics, mitochondrial DNA (mtDNA) maintenance, and mitochondrial dynamics. The variations in the *FBXL4* gene can give rise to encephalomyopathy mitochondrial DNA depletion syndrome-13 (MTDPS13) characterized by the reduction of mtDNA copy number, leading to deficiencies in mitochondrial functions, which is a serious and rare autosomal recessive genetic disorder. Patients with *FBXL4* variations are usually diagnosed due to the emergence of symptoms in the early stages of life. Commonly observed are lactic acidemia, developmental retardation, and hypotonia. A portion of patients may be accompanied by comorbidities such as cardiovascular diseases, epilepsy, ophthalmopathy, hearing impairment, and movement disorders. Currently, there have been no reported cases of prenatal diagnosis for *FBXL4* gene variations. Here, we report for the first time the prenatal diagnosis of a fetus with a compound heterozygous mutation in the *FBXL4* gene (NM_012160.5: c.1288C>T, p. Arg430* and c.518_523del, p. Glu173_Leu175delinsVal) by trio-WES, the nonsense mutation (c.1288C>T) was reported only once in an unrelated individual and no detailed clinical phenotype; the deletion mutation (c.518_523del) has not been reported yet. Additionally, we monitor prenatal phenotypes of fetus at different stages of pregnancy using ultrasound and magnetic resonance imaging (MRI), present prenatally with nuchal translucency (NT) thickening and progressive brain developmental abnormalities. Our report indicates that the application of trio whole exome sequencing (trio-WES) and imaging monitoring can facilitate prenatal diagnosis of *FBXL4* gene-related MTDPS13, and this will modify the decision-making process for couples with *FBXL4* variations.

## 1 Introduction

Mitochondria are the energy-producing structures in cells and the primary site for aerobic respiration. Mitochondrial autophagy is a process by which damaged, dysfunctional, or excessive mitochondria are selectively processed through autophagy, playing a vital role in maintaining cellular equilibrium (Aman et al., 2021). F-box and leucine-rich repeat protein 4 (FBXL4) is a member of the F-box protein family, and a protein located on the outer mitochondrial membrane that participates in regulating mitochondrial autophagy (Cao et al., 2023). The human genome encodes 69 F-box proteins, which are regarded as substrate receptors to facilitate the recognition of the Skp1-Cul1-F-box (SCF) E3 ubiquitin ligase complex. The SCF-FBXL4 E3 ubiquitin ligase complex is situated on the outer mitochondrial membrane, constitutively mediates the ubiquitination and degradation of BNIP3L/NIX and BNIP3 mitochondrial autophagy receptors to suppress mitochondrial autophagy. The post-translational regulation of BNIP3L and BNIP3 is disrupted in mitochondrial DNA depletion syndrome 13 (MTDPS13), a multisystem disorder disease caused by variations in the *FBXL4* gene (Cao et al., 2023). MTDPS13 is a rare autosomal recessive disease induced by biallelic mutations in the F-box and leucine-rich repeat (LRR) protein 4 gene (*FBXL4*) (Gai et al., 2013), characterized by increased mitochondrial autophagy and mitochondrial DNA (mtDNA) depletion in *FBXL4* knockout mice and patient fibroblasts (Alsina et al., 2020).

Since its first description in 2013 (Bonnen et al., 2013; Gai et al., 2013), a total of 59 pathogenic variations in *FBXL4* have been identified in 112 patients with autosomal recessive inheritance. These variations occur through homozygosity or compound heterozygosity (Gao et al., 2024). Our case will be the 113rd reported case and the first prenatal diagnosis. Among them, the deletion mutation (c.518_523del) is a novel variation in *FBXL4* gene. Patients with *FBXL4* variations are typically diagnosed due to the manifestation of symptoms in the early stages of life. The associated symptoms of MTDPS13 can vary considerably among different patients, *FBXL4* variations tend to exert more severe influences on energy-demanding organs such as the brain, heart, muscles, and the renal system. Commonly reported symptoms encompass hypotonia, neurodevelopmental delay, lactic acidemia, microcephaly, hyperammonemia, and growth failure. Additionally, a series of less frequent manifestations have been identified, including extensive neurological, ophthalmological, cardiac, gastrointestinal, urogenital, and immunological manifestations. Special facial features have been observed in some affected individuals. Abnormalities detected in patients undergoing brain magnetic resonance imaging (MRI) include white matter abnormalities, diffuse cerebral atrophy, basal ganglia abnormalities, ventricular dilation, periventricular cysts, brainstem abnormalities, thinning of the corpus callosum, and arachnoid cysts (El-Hattab et al., 2017; Gao et al., 2024). The prognosis of MTDPS13 patients is extremely unfavorable, with a high mortality rate. The causes of death include sepsis, severe acidosis, pneumonia, and cardiopulmonary failure (El-Hattab et al., 2017).

There is no reported prenatal diagnostic of MTDPS13 linked to *FBXL4*. For the neonates died with *FBXL4* variations, the review of prenatal phenotypes show periventricular cysts, periventricular echogenicity, ventriculomegaly, thin corpus callosum, mega cisterna magna, and large cavum (Saini et al., 2022), polyhydramnios and cerebellar atrophy (van Rij et al., 2016a). Therefore, prenatal diagnostic for *FBXL4* variants is important, which will help pregnant’ women making decision. This case will report the prenatal diagnostic of MTDPS13 linked to *FBXL4* detected by MRI and trio Whole Exome Sequencing (trio-WES) and its detailed prenatal phenotypes.

## 2 Fetal phenotype

The prenatal diagnostic center received a referral for a 32-year-old pregnant woman. The obstetric history of pregnant woman includes two pregnancies and one birth. The couple is not relatives. Besides, the family history was non-contributory. The pregnant’s previous ultrasound had no significant findings except increased nuchal translucency (NT: 3.7 mm). At 22^+3^ weeks of gestation, the pregnant woman underwent amniocentesis for copy number variation sequencing (CNV-seq) of the fetal amniotic fluid, which did not reveal any CNVs or chromosome aneuploidies that may lead to NT thickening. After receiving thorough consultation from a geneticist, the pregnant woman agreed to perform trio-WES on the genomic DNA extracted from the fetal amniotic fluid and parental peripheral blood, which identified a compound heterozygous variant in the *FBXL4* gene. After reviewing literature and databases, no prenatal report of NT thickening related to *FBXL4* gene was found, which may be associated with prenatal phenotypes such as abnormal brain development (including ventricular dilatation, thin corpus callosum, cerebellar hypoplasia, etc.) and polyhydramnios (Saini et al., 2022; van Rij et al., 2016b).

**FIGURE 1:**
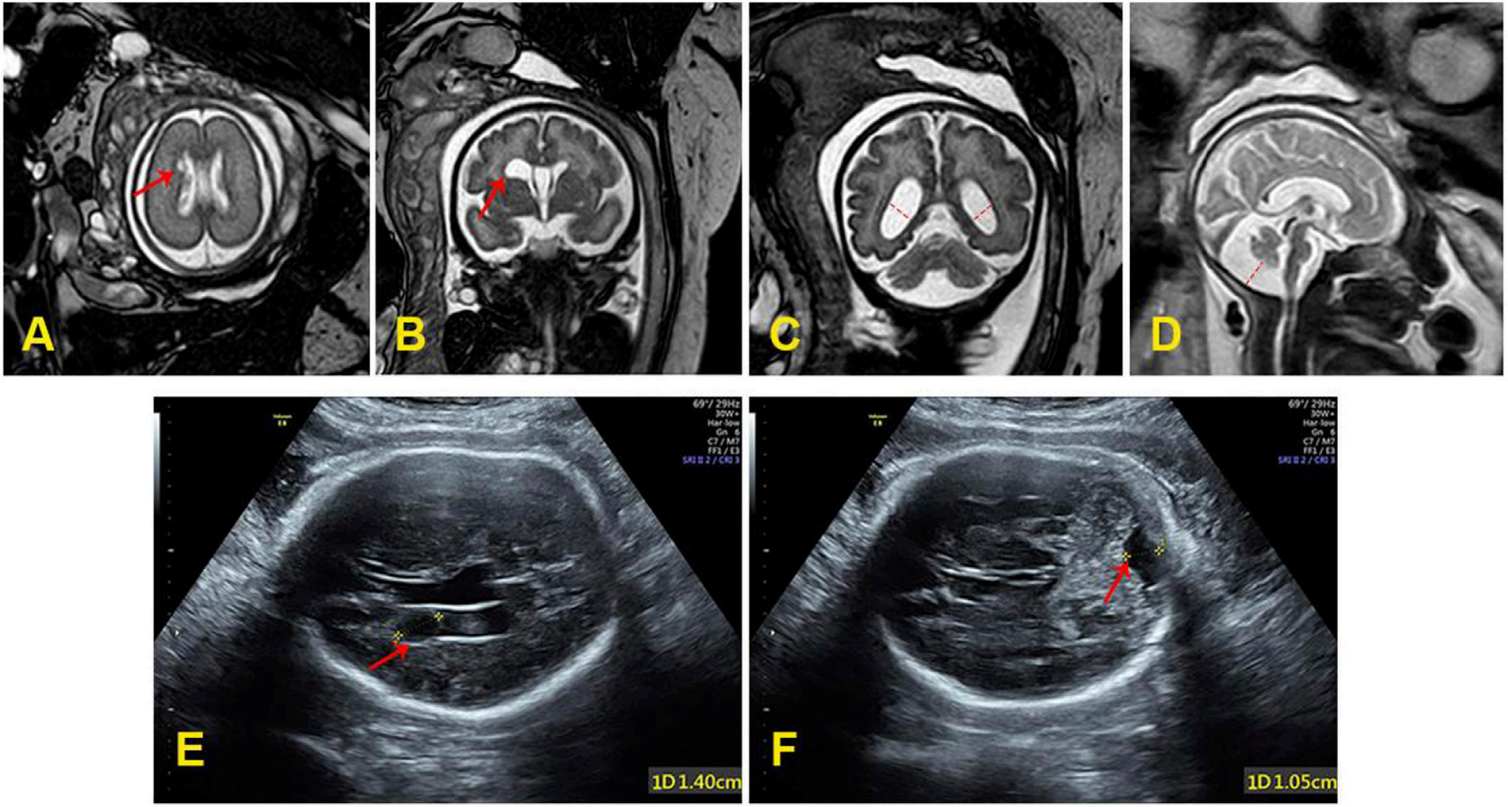
**(A)** The subependymal cyst (0.5 cm × 0.3 cm) at 26+4 weeks of gestation. **(B)** The subependymal cyst (1.62 cm × 1.26 cm × 0.88 cm) at 35 weeks of gestation. **(C)** The bilateral lateral ventricle dilatation (left: 1.19 cm, right: 1.01 cm) at 35 weeks of gestation. **(D)** The enlarged posterior fossa (1.37 cm) at 35 weeks of gestation. **(E)** The subependymal cyst (1.4 cm) at 35+6 weeks of gestation. **(F)** The posterior fossa was close to 1.0 cm at 35+6 weeks of gestation.

In order to further evaluate the prognosis of the fetus and the pathogenicity of *FBXL4* gene variations, the geneticist recommended conducting fetal brain MRI. The targeted fetal brain MRI at 26^+4^ weeks of gestation showed a subependymal cyst located on the left side of the anterior horn of the lateral ventricle (Figure 1A). Reexamination MRI performed at 35 weeks of gestation showed the subependymal cyst had enlarged compared to the previous examination (Figure 1B), along with bilateral lateral ventricle dilatation (Figure 1C) and the enlarged posterior fossa (Figure 1D). The targeted ultrasound at 35^+6^ weeks of gestation also indicated a subependymal cyst (Figure 1E), as well as a fetal posterior fossa measuring approximately 1.0 cm (Figure 1F).

## 3 Diagnostic method

The trio-WES analysis was performed using an Illumina NovaSeq6000 platform. Sequencing reads were aligned to the reference human genome GRCh38/hg38 with Burrows-Wheeler Aligner (v0.7.17). Functional annotation was performed using the ENLIVEN variants interpretation system. Suspected variants identified through Trio-WES were confirmed by Sanger sequencing (c.1288C>T-Forward sequence: TCA​GAG​TAG​TCA​GAA​GGC​ATC​A; c.1288C>T-Reversed sequence: TGA​ATT​GTC​TTG​CAG​CCA​CTT, c.518_523del-Forward sequence: GCA​CTG​GCT​TGT​CCT​TCA​C; c.518_523del-Reversed sequence: AGG​TCT​GTG​GCA​TTT​GGT​TT).

## 4 Genetic results and pregnancy outcomes

A compound heterozygous variation in the *FBXL4* gene (NM_012160.5: c.1288C>T, p. Arg430* and c.518_523del, p. Glu173_Leu175delinsVal) was detected by trio-WES (Table 1). The c.1288C>T variation was inherited from unaffected father. This variation has been documented in the HGMD database and was identified in patient diagnosed with MTDPS13. However, no specific phenotypes description was provided in the article (Smedley et al., 2021).

**TABLE 1:** Genetic findings.

Procedure (gest age)	Direct/culture	Performed test	Secondary confirmatory test	Gene name. (REFSEQ)	Known disease (OMIM)	Variants	ACMG Classification (Criteria Applied)^a^	Inheritance and Zygosity	Interpretation
Amniotic fluid (22^+3^weeks)	Direct	Trio Whole exome sequencing (trio-WES)	Sanger sequencing	*FBXL4* (NM_012160.5)	Mitochondrial DNA depletion syndrome 13 (encephalomyopathic type); MTDPS13 (OMIM: 615471)	Allele 1: c.1288C>T (p. Arg 430*)	likely pathogenic (PVS1, PM2)	Paternally Inherited	Causative
						Allele 2: c.518_523del (p. Glu173_Leu175delinsVal)	likely pathogenic (PM2, PM3, PM4)	maternally inherited	

^a^ ACMG Classification: Standards and guidelines for the interpretation of sequence variants: a joint consensus recommendation of the American College of Medical Genetics and Genomics and the Association for Molecular Pathology (Richards et al., 2015).

**FIGURE 2:**
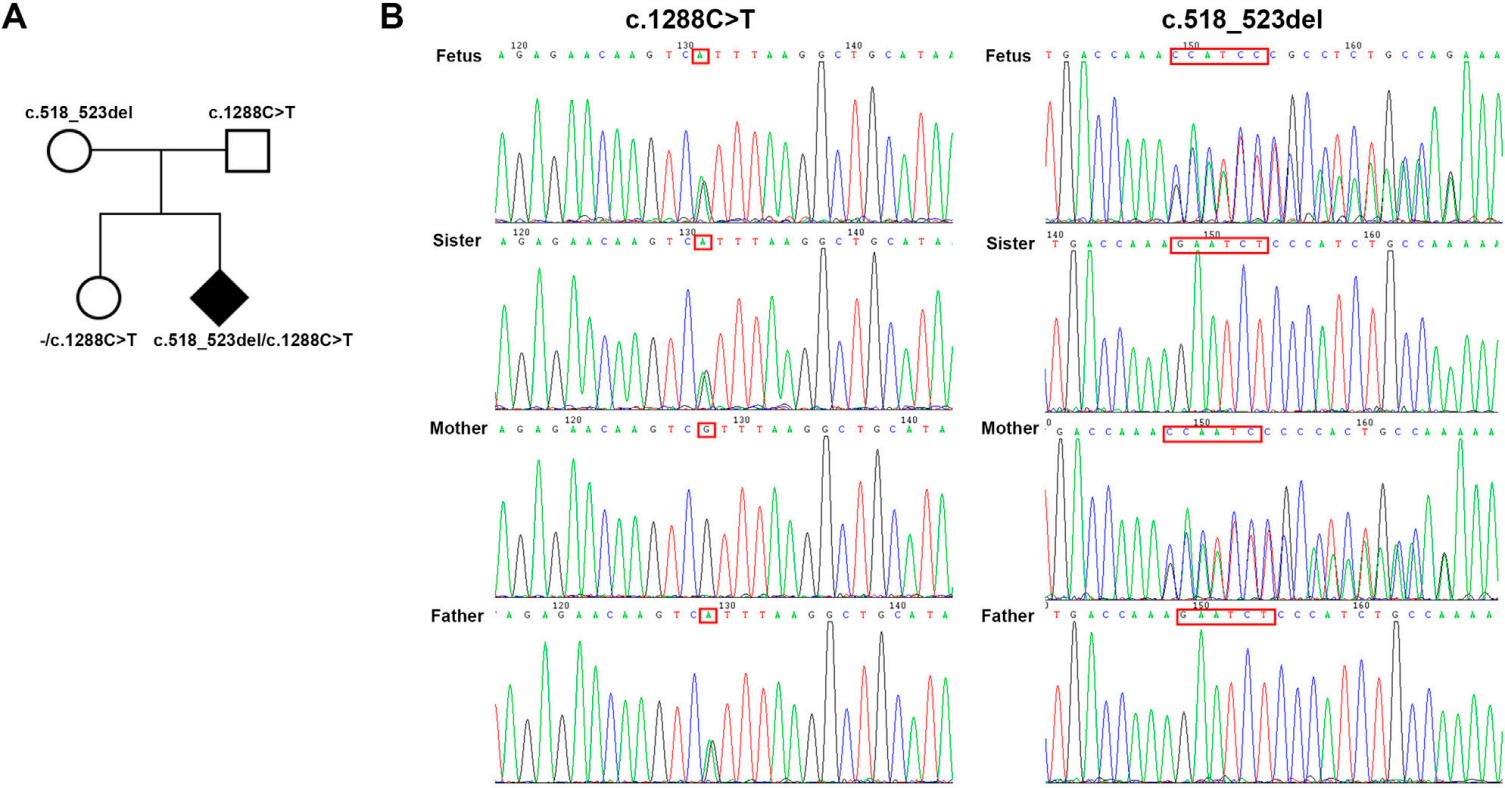
**(A)** Family tree diagram. **(B)** Sanger sequencing verification.

The novel c.518_523del variation was inherited from the unaffected mother. Following sanger sequencing verification, the full sister of the fetus, who does not exhibit any abnormal phenotype, only inherits the father’s nonsense variation (Figure 2B). The remaining verification results are consistent with trio-WES (Figure 2A).

Finally, the family opted for pregnancy termination following a comprehensive multidisciplinary consultation.

## 5 Disscusion

The NT thickening of the fetus in the early stage of pregnancy is a common phenotypic manifestation of chromosomal abnormalities. Besides, it is associated with various fetal malformations, dysplasias, deformations, disruptions, and an increased risk of adverse perinatal outcomes caused by genetic syndromes (Souka et al., 2005). Our case represents the first report of a compound heterozygous variation in the *FBXL4* gene being identified in cases of NT thickening during early pregnancy. Notably, we detected subarachnoid cysts in the fetus at mid-pregnancy through targeted brain MRI, with cyst enlargement, ventriculomegaly, and enlargement of the posterior fossa cistern in the late pregnancy. The prenatal phenotypes of fetus brain verified the trio-WES result, *FBXL4* variants can lead to severe influence on brain (El-Hattab et al., 2017), which suggests that *FBXL4* is the pathogenic gene for the fetus. Besides, this indicates that the phenotypes of most patients with severe encephalopathy due to MTDPS13 have already emerged prenatally but have been overlooked as they were not detected during routine prenatal examinations. Therefore, in the limited prenatal diagnostic window period, diagnostic methods should be fast and comprehensive. With the informed consent of the pregnant woman and her family, for fetuses with prenatal phenotypes such as NT thickening (NT > 3.5 mm, especially), it is recommended to consider simultaneous chromosome and genetic testing (trio-WES) and strengthen follow-up imaging monitoring. Additionally, detailed family history queries might be necessary. The comprehensive testing can aid in decision-making for the pregnant women, offering more evidence.

**FIGURE 3:**
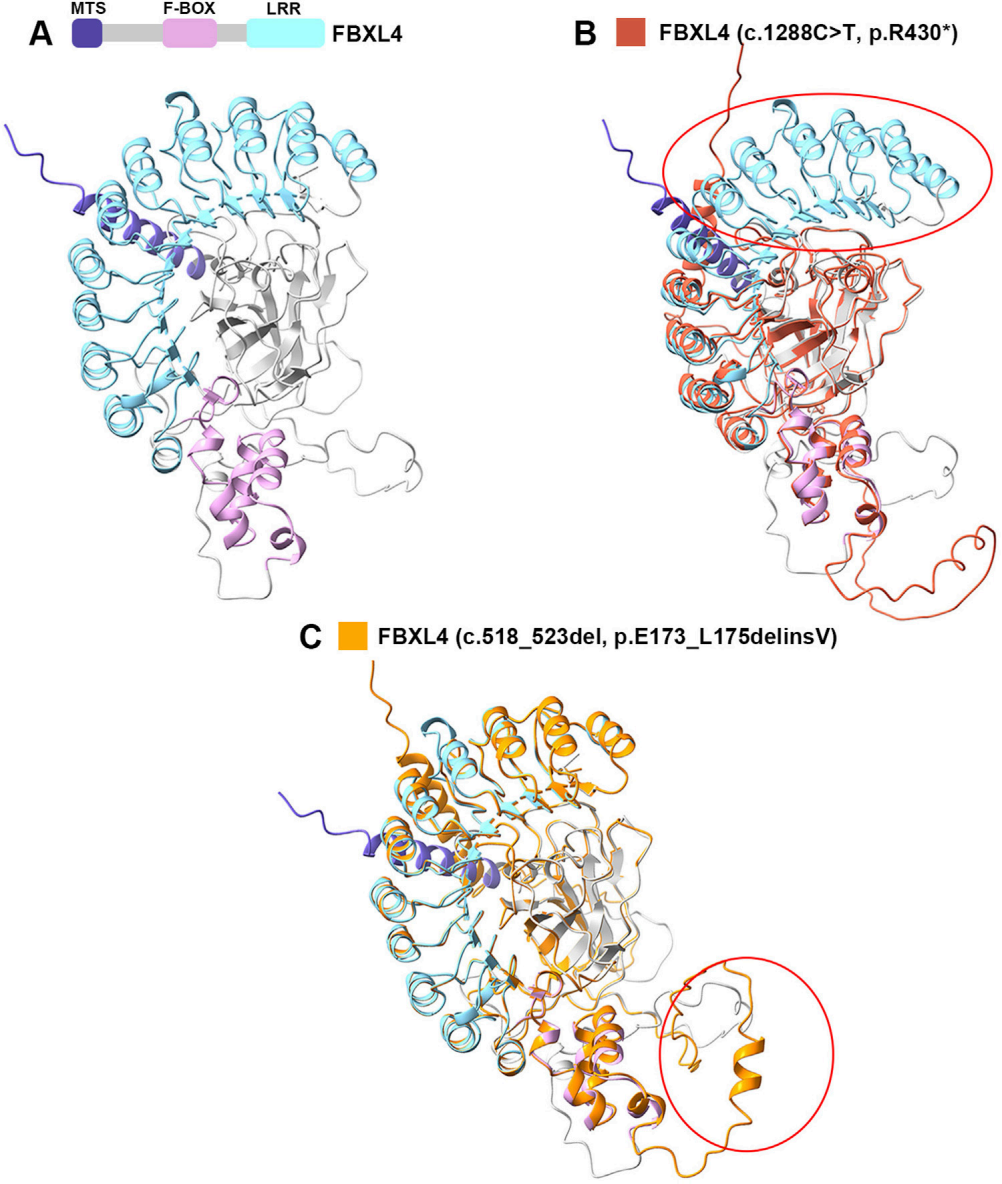
The schematic of FBXL4 and FBXL4’s variants predicted structure by AlphaFold2. **(A)** Schematic of FBXL4 domain organization and predicted structure. **(B)** The comparison between the structures of FBXL4 with its nonsense variation (c.1288C>T, p. R430*). **(C)** The comparison between the structures of FBXL4 with its deletion variation (c.518_523del, p. E173_L175delinsV).

*FBXL4* is 621 amino acids in length and comprises an N-terminal mitochondrial targeting sequence (MTS) (amino acids 1–26), an F-box domain (amino acids 277–332), and 9 leucine-rich repeat (LRR) domain (amino acids 340–609) (Figure 3A) (Bonnen et al., 2013; Gai et al., 2013). Two mutations of *FBXL4* were detected in this fetus: a nonsense variation c.1288C>T (p. Arg430*) and a deletion variation c.518_523del (p. Glu173_Leu175delinsVal). The nonsense variation resulted in a reduction of 191 amino acids in *FBXL4* and disrupted the LRR sequence (Figure 3B). The deletion variation caused change near F-BOX in the protein structure (Figure 3C). These structural changes may have severe influence on the function of FBXL4 protein. Additionally, relevant studies have indicated that the phenotypes of nonsense and deletion variations are more severe compared to missense variations (El-Hattab et al., 2017). The average lifespan of patients is 4 years, with a median of 2 years (El-Hattab et al., 2017). The fetus in this case also exhibited severe brain structural developmental abnormalities prenatally. Therefore, the pregnant couple chose to terminate the pregnancy.

In conclusion, our case is the first case of prenatal diagnosis of MTDPS13, provides a detailed description of the prenatal phenotype during pregnancy, which will improve decision-making for couples in ongoing pregnancies with *FBXL4* variation.

## Data Availability

The datasets presented in this study can be found in online repositories. The names of the repository/repositories and accession number(s) can be found in the article/supplementary material.
